# Rendezvous between ambulances and prehospital physicians in the Capital Region of Denmark: a descriptive study

**DOI:** 10.1186/s13049-022-01040-2

**Published:** 2022-10-11

**Authors:** Roselil Oelrich, Julie Samsoee Kjoelbye, Oscar Rosenkrantz, Charlotte Barfod

**Affiliations:** 1 Copenhagen Emergency Medical Services, University of Copenhagen, Telegrafvej 5, 2750 Copenhagen, Capital Region of Denmark Denmark; 2 Department of Clinical Medicine, University of Copenhagen, Copenhagen, Denmark

**Keywords:** Prehospital, Emergency physician, Rendezvous, Unclear problem, Dispatch criteria, Ambulance

## Abstract

**Background:**

In a two-tier Emergency Medical Services response system with ambulances and physician-staffed rapid response vehicles, both units are ideally dispatched simultaneously when a physician is needed. However, when advanced resources are dispatched secondarily, a meeting point (rendezvous) is established to reduce time to advanced care. This study aims to assess the extent of rendezvous tasks, patient groups involved and physician contribution when rendezvous is activated between the primary ambulances and rapid response vehicles in the Capital Region of Denmark.

**Methods:**

We analysed prehospital electronic patient record data from all rendezvous cases in the Capital Region of Denmark in 2018. Variables included the number of times rendezvous was activated, patient demographics, dispatch criteria, on-scene diagnosis, and prehospital treatment.

**Result:**

Ambulances requested rendezvous 2340 times, corresponding to 1.3% of all ambulance tasks and 10.7% of all rapid response vehicle dispatches. The most frequently used dispatch criterion was *unclear problem* n = 561 (28.8%), followed by *cardiovascular* n = 439 (22.5%) and *neurological* n = 392 (20.1%). The physician contributed with technical skills like medication n = 760 (39.0%) and advanced airway management n = 161 (8.3%), as well as non-technical skills like team leading during advanced life support n = 152 (7.8%) and decision to end futile treatment and death certificate issuance n = 73 (3.7%).

**Conclusion:**

Rendezvous between ambulances and physician-staffed rapid response vehicles was activated in 1.3% of all ambulance cases corresponding to 10.7% of all RRV dispatches in 2018. The three largest patient groups in rendezvous presented *cardiovascular*, *neurological,* and *respiratory problems*. The prehospital physician contributed with technical skills like medication and advanced airway management as well as non-technical skills like team leading during advanced life support and ending futile treatment. The high percentage of dispatch criterion *unclear problem* illustrates the challenge of precise dispatch and optimal use of prehospital resources. Therefore, it seems necessary to have a safe and rapid rendezvous procedure to cope with this uncertainty.

## Background

The Copenhagen Emergency Medical Services (EMS) handles call-taking and dispatching related to acute life-threatening injuries and illnesses in the Capital Region of Denmark through the countrywide emergency telephone number 112 [1]. Each year, specially trained nurses or paramedics handle around 130,000 calls using criteria based dispatch [2, 3]. In most cases, an ambulance is dispatched as the primary response, but in specific potentially time-critical conditions, a physician-staffed rapid response vehicle (RRV) is dispatched simultaneously [4]. However, in some cases, the ambulance crew will identify the need for physician assistance after initial patient contact. Thus, it is critical to establish a meeting point (rendezvous) rapidly and safely between the ambulance crew and physician, preferably while the ambulance moves towards the receiving hospital. The RRVs are primarily used as advanced support to ambulances in potentially time-critical conditions but also assist the ambulances in subacute tasks like mental institution admission and death certificate issuance. This study aims to assess the extent of rendezvous tasks, patient groups involved and physician contribution when rendezvous is activated between the primary ambulances and RRVs in the Capital Region of Denmark.

## Methods

### Study design

The study is a retrospective, observational study on rendezvous cases at the Copenhagen EMS, covering the capital region of Denmark in 2018.

### Setting

The Capital Region of Denmark covers an area of 2555.4 square kilometres with a population of 1,8 million [5]. The Copenhagen EMS provides prehospital critical care and transport in medical emergencies. Denmark and other European countries use two-tiered emergency medical systems with ambulances staffed by emergency medical technicians or paramedics and non-transporting RRVs staffed by a physician and a paramedic [6]. In Denmark, the physician is a consultant-level anesthesiologist with additional training in prehospital critical care [7]. The region's five RRVs are, together with physician-staffed helicopters [8], the most advanced part of the prehospital system, primarily used for potentially life-threatening conditions like cardiac arrest, severe trauma, or difficult airway conditions. However, RRVs also assist in interhospital transportation, mental institution admission, death certificate issuance and other primarily subacute tasks, as well as supervision and teaching of the ambulance crews [9]. After each case, prehospital observations, interventions applied in the field and patient data are documented in a prehospital electronic patient record (PEPR) by the ambulance and RRV crews.

### Criteria based dispatch

A criteria based dispatch system guides emergency medical dispatchers in assessing severity and determining response, including the urgency, competencies, and resources dispatched. The system, Danish Index for Emergency Care, is based on the Norwegian edition and is used nationwide [3, 10]. All incoming calls are categorised based on symptom-specific criteria with the possibility of labelling the case *unclear problem*. If specific criteria are met, the dispatcher will send an RRV simultaneously with the ambulance as the primary response.

### Participants

We screened all cases where the ambulance crew requested rendezvous with an RRV after patient contact on-scene, from the 1st of January to the 31st of December 2018. All cases where the RRV and ambulance met, either on-scene or on the way to the hospital, were included. Cases were excluded if rendezvous were not obtained, if extensive data was missing, if the ambulance crew cancelled the RRV request, and in cases with no available RRV.

### Data collection, variables, and definitions

Data were collected from the PEPR and the local Computer Assisted Dispatch database. Data of interest were patient age and sex, dispatch criterion, on-scene assessment and interventions performed by the physician. The last dispatch criteria given by the dispatcher were used to categorise the cases according to the Danish Index of Emergency Care. All cases in the category *unclear problem* were further investigated and categorised by the author Oelrich, R. The categories used corresponded to the dispatch criteria categories. They were decided upon using documentation from the RRV physicians’ on-scene assessments.

Physician interventions were categorised into two main groups: *technical* and *non-technical* skills. Interventions were non-exclusive, i.e., more than one intervention could be registered per case. Interventions were collected from the PEPR, where RRV physicians fill out interventions by ticking boxes or documenting them as notes.

The study group defined technical interventions as using an instrument or medications to treat a patient's symptoms and non-technical interventions as interventions, which primarily were communicative and coordinative like *team leading during advanced life support*, *decision to end futile treatment*, *death certificate issuance*, *admission to a mental institution* and coordinating with police and fire brigade in the role as *medical on-scene commander.*

## Results

**Fig. 1:**
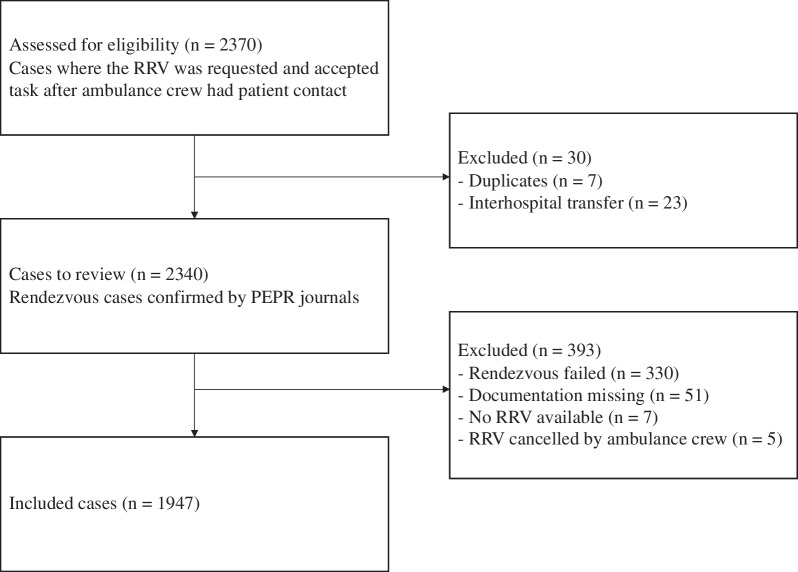
Flowchart of inclusion and exclusion resulting in 1947 included cases

During the study period, ambulances were dispatched 177,109 times, of which 82,253 were with lights and sirens. The RRVs were dispatched 19,539 times simultaneously with the ambulance as primary response and secondarily on ambulance request in 2340 cases. Thus, rendezvous was activated in 1.3% of all ambulance tasks, corresponding to 10.7% of all RRV dispatches. Data showed that ambulance crews requested rendezvous in 2370 cases. Of these, 30 were excluded (7 duplicates and 23 interhospital transfers) (Fig. 1).

Thus, the study group reviewed 2340 potential rendezvous cases. Prehospital electronic patient records were then obtained and extracted for evaluation. Rendezvous failed in 330 cases, RRVs were unavailable in 7 cases, cancelled by the ambulance crew 5 times, and 51 had missing data. In total, 393 cases were excluded; thus, rendezvous was achieved, and data available in 1947 of 2340 requested rendezvous cases (83.2%).

### Demographic

**Fig. 2:**
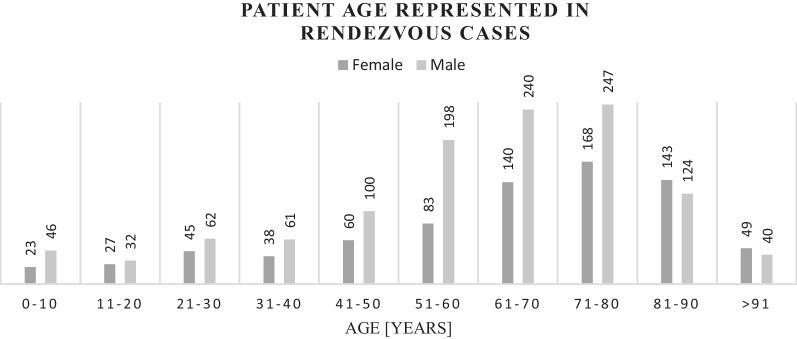
Patient age and sex in rendezvous cases. Missing data: sex n = 14, age n = 18

A total of 1947 cases involving 1947 patients included 780 females (40.1%),1153 males (59.2%) and 14 cases with missing data (0.7%). The median age was 61 years (IQR 50–77) (Fig. 2).

### Dispatch criteria

The included cases were categorised by the emergency medical dispatchers’ dispatch criteria, including the category *other* [10] containing *fever, gynaecology, burn injury, consultation*, *social help, urinary tract complications, prescription* and *ear, nose and throat problems* (Table 1)*.*

**Table 1:** Dispatch criteria from a total of 1947 rendezvous cases

Dispatch criteria	Quantity (%)
Unclear problem	561 (28.8)
Cardiovascular	439 (22.5)
Neurological	392 (20.1)
Respiratory	242 (12.4)
Trauma/injury	179 (9.2)
Alcohol, drugs and poisoning	49 (2.5)
Abdominal pain	32 (1.6)
Psychiatry	20 (1.0)
Paediatrics	13 (0.7)
Other	20 (1.0)

The criterion most frequently used by the dispatchers, resulting in rendezvous, was *unclear problem*, which amounted to n = 561 (28.8%), followed by *cardiovascular* n = 439 (22.5%), *neurological* n = 392 (20.1%), *respiratory* n = 242 (12.4%), *trauma/injury* n = 179 (9.2%), *alcohol, drugs and poisoning* n = 49 (2.5%), *abdominal pain* n = 32 (1.6%), *psychiatry* n = 20 (1.0%), *paediatrics* n = 13 (0.7%) and *other* n = 20 (1.0%).

### On-scene assessment by the RRV physician

Cases categorised as *unclear problem* were further reviewed for on-scene assessment and diagnosis given by the physician. The most frequent diagnoses were *cardiovascular* (52.2%), followed by *neurological* (18.5%), *respiratory* (11.9%), *alcohol, drugs, and poisoning* (5.0%), *trauma/injury* (4.3%), *psychiatry* (4.3%), and *abdominal pain* (1.1%). The group *Other* (2.7%) included *burn injury, ear, nose and throat problems* and *gynaecology*. Thus, accounting for a similar distribution pattern of criteria given by the dispatchers for the total rendezvous population. These on-scene assessments revealed critical medical conditions, including cardiac arrest, other critical heart diseases, critical neurological conditions and respiratory distress (Table 2).

**Table 2:** Diagnoses in the category *unclear problem* based on physician on-scene assessment

Diagnoses from the dispatch criteria *unclear problem*	Quantity (%)
Cardiovascular	293 (52.2)
Neurological	104 (18.5)
Respiratory	67 (11.9)
Alcohol, drugs, and poisoning	28 (5.0)
Trauma/injury	24 (4.3)
Psychiatry	24 (4.3)
Abdominal pain	6 (1.1)
Other	15 (2.7)

### RRV interventions

In total, 1081 technical interventions and 263 non-technical interventions were registered. More than one type of intervention could be registered per patient. The most frequent technical interventions were administration of *medicine* n = 760 (39.0%), advanced *airway management* including intubation n = 161 (8.3%) and *ultrasound* n = 86 (4.4%). The non-technical interventions were *team leading during advanced life* n = 152 (7.8%), decision to *end futile treatment* and *death certificate issuance* n = 73 (3.7%), *admission to a mental institution* n = 26 (1.3%) and *medical on-scene commander* coordinating with police and fire department n = 12 (0.6%) (Table 3).

**Table 3:** Technical and non-technical interventions performed by the RRV physician in rendezvous cases. Percentage of all rendezvous cases

Technical interventions	N (%)	Non-technical interventions	N (%)
Medicine	760 (39.0)	Team leading during advanced life support	152 (7.8)
Intubation and- Supraglottic Airway	108 (5.5)	End futile treatment and death certificate issuance	73 (3.7)
Ultrasound	86 (4.4)	Admission to a mental institution	26 (1.3)
Intraosseous and intravenous access	38 (2.0)	Medical on-scene commander	12 (0.6)
Continuous positive airway pressure-(CPAP)/ Assisted spontaneous breathing	32 (1.6)		
Suctioning	21 (1.1)		
Direct Current shock, External pacing and ICD/pacemaker handling	18 (0.9)		
External cardiac compression	12 (0.6)		
Transfusion (Dried Plasma)	6 (0.3)		

### Medicine

Medicine was administrated by the ambulance crew or the RRV physicians. A physician administered medication in 760 (39.0%) cases. More than one type of medicine could be registered per patient. The medication most frequently administrated after RRV arrival was *fentanyl* n = 239 (12.3%), *ondansetron* n = 125 (6.4%), *nitroglycerin* n = 81 (4.2%), *adrenaline* n = 74 (3.8%), *propofol* n = 73 (3.7%), *suxamethonium* n = 48 (2.5%) and *adenosine* n = 44 (2.3%). Apart from *propofol* and *suxamethonium*, used for endotracheal intubation, *adenosine* was the most used drug not available to the ambulance crew (Table 4).

**Table 4:** Medicine administered by the RRV physician in rendezvous cases. Percentage of total medicine administrations

Medicine available for Ambulance crews	N (%)	Medicine available only for RRV physicians	N (%)
Fentanyl	239 (12.3)	Propofol	73 (3.7)
Ondansetron	125 (6.4)	Suxamethonium	48 (2.5)
Nitroglycerin	81 (4.2)	Adenosine	44 (2.3)
Adrenaline	74 (3.8)	Methylprednisolone	35 (1.8)
Furosemide	67 (3.4)	S-Ketamine	31 (1.6)
Heparin	67 (3.4)	Tranexamic acid	31 (1.6)
Amiodaron	63 (3.2)	Ephedrine	25 (1.3)
Midazolam	61 (3.1)	Ipratropium	23 (1.2)
Natriumchlorid	58 (3.0)	Rocuronium	22 (1.1)
Diazepam	55 (2.8)	Phenylephrine	21 (1.1)
Atropine	33 (1.7)	Alfentanil	14 (0.7)
Naloxone	23 (1.2)	Metoprolol	11 (0.6)
Clemastine	16 (0.8)	Flumazenil	9 (0.5)
Salbutamol	12 (0.6)	Benzylpenicillin	7 (0.4)
Glucose 5%	6 (0.3)	Calcium chloride	6 (0.3)
Glucagon	1 (0.1)	Magnesium sulfate	6 (0.3)
Ticagrelor	1 (0.1)	Isoprenaline	4 (0.2)
		Terbutaline	2 (0.1)
		Other antibiotics	2 (0.1)
		Ceftriaxone	1 (0.1)
		Olanzapine	1 (0.1)

## Discussion

This study identified 2340 cases requesting rendezvous with RRV, 1.3% of all ambulance tasks in the Capital Region of Denmark in 2018. In these cases, the need for an RRV was not identified during the emergency call, or the situation worsened after first contact. The RRV dispatch is based on guidelines, and if specific parameters are met, for instance, cardiac arrest or severe trauma, they are dispatched simultaneously with the ambulance [10]. In cases of rendezvous, patients’ conditions are often more unclear during the emergency call, and therefore an RRV is not dispatched primarily.

The most frequent dispatch criterion resulting in rendezvous cases was *unclear problem*, representing 28.8% of all cases. The category *unclear problem* revealed severe medical conditions like cardiac arrest, other critical heart diseases, acute neurological conditions, and severe respiratory distress. The challenge and difficulty of an accurate dispatcher assessment are well known. A study by Møller et al. investigating calls labelled *unclear problem* showed that language difficulties, age of caller and time of day are factors impacting why calls are categorised as *unclear problem* [11]. Further, the high number of unclear cases might reflect the difficulty of telephone dispatch with the tools and technology available today. A Norwegian study by Johnsen et al. showed that using video in emergency calls could improve the dispatchers’ understanding of the situation [12]. Another study looking at video transmitting from the caller directly to the dispatcher found that live video can be used to provide dispatchers with critical information [13].

In a perfect dispatch triage process, the need for rendezvous in a two-tiered EMS system would be almost obsolete. However, emergency medical dispatch is complex, and medical emergencies can escalate after contact with the caller. Therefore, it is essential to establish an efficient and safe procedure for rendezvous when the primary ambulance requests assistance from a higher level of competencies. The guidelines for the ambulance crew to request assistance from the RRV crew were situations where the patient’s vital signs were critical, condition with a risk of rapid deterioration (for instance ST- elevation infarction), or the mechanism of injury was potentially critical, for instance, fall from heights or penetrating trauma [4].

The professional cooperation between ambulance and RRV crew includes both technical and non-technical skills. Administration of medicine was the most frequently used technical intervention, with 39.0% of rendezvous cases. The ambulance crew either did not have the medicine or had limited dosage or indication in using specific drugs. The ambulance crew have the option to call the RRV for approval to exceed the dosage limit for a specific medication. The most frequently used medications were *fentanyl*, *ondansetron*, and *nitroglycerin*, all available to the ambulance crew but in limited dosages. *Propofol* and *suxamethonium*, used for sedation and relaxation in advanced airway management, are unavailable to the ambulance crew. Endotracheal intubation was the third most used technical intervention in rendezvous. In Denmark, the ambulance crew are trained to use nasopharyngeal airway, oropharyngeal airway, and supraglottic devices like laryngeal masks but not to perform endotracheal intubation. If there is a need for endotracheal intubation, it is performed by the RRV physician. These examples illustrate that the reason for requesting rendezvous is highly dependent on the competence level of the ambulance crew.

Close collaboration between ambulance and RRV crew contributes to a good and safe learning environment as well as professional development in critical and less critical situations. In some cases, physician involvement is determined by law, for instance, in the use of coercion in the admission of psychiatric patients or the decision to end futile treatment. In cardiac arrest, the physician acts as team leader and contributes with advanced medical competencies and advanced airway skills. Furthermore, the physician is essential in stabilising and escorting the patient if return of spontaneous circulation is achieved. In major incidents where police and fire departments are present, the physician is part of the command-and-control structure and coordinates the prehospital effort. In all cases of rendezvous, the RRV crew contributes with critical decision-making, clinical assessment and advice, as well as teaching and supervision of the ambulance crew.

## Limitations

Due to this study’s retrospective nature, it is not possible to determine the precise reason why the ambulance crew requested rendezvous and the study design does not allow us to conclude if rendezvous was sent to the right patients. Therefore, the study cannot identify any possible beneficial effects of rendezvous. Further, when a diagnosis was missing in the PEPR, the authors determined which category was most appropriate based on the physician´s notes. Lastly the generalisability of results depends on the local level of competencies, as well as the prehospital setting. This includes guidelines, culture, resources available and the typical distance from scene to hospital, which might influence the need for rendezvous.

## Conclusion

Rendezvous between ambulances and physician-staffed rapid response vehicles was activated in 1.3% of all ambulance cases corresponding to 10.7% of all RRV dispatches in 2018. The three largest patient groups in rendezvous presented *cardiovascular*, *neurological,* and *respiratory problems*. The prehospital physician contributed with technical skills like medication and advanced airway management as well as non-technical skills like team leading during advanced life support and ending futile treatment. The high percentage of dispatch criterion *unclear problem* illustrates the challenge of precise dispatch and optimal use of prehospital resources. Therefore, it seems necessary to have a safe and rapid rendezvous procedure to cope with this uncertainty.

## Data Availability

The datasets analysed during the current study are available from the corresponding author on request.

## Appendix

DISPATCH CRITERIA TABLE* Danish Index for Emergency care

**Table:**

**Criterion** 01 unconscious adult after puberty **Criterion** 02 unconscious child in puberty **Criterion** 03 Foreign body in airway **Criterion** 04 Disaster—big accident **Criterion** 05 Pre ordered case **Criterion** 06 unclear problem **Criterion** 07 Allergic reaction **Criterion** 08 Bleeding—non traumatic **Criterion** 09 Burn injury—electricity damage **Criterion** 10 Chest pain—heart disease **Criterion** 11 Diabetes **Criterion** 12 Drowning **Criterion** 13 Diving accident **Criterion** 14 Animal or insect bite **Criterion** 15 Fever **Criterion** 16 Intoxication (Children) **Criterion** 17 Birth **Criterion** 18 Gynaecology—pregnancy **Criterion** 19 Headache	**Criterion** 20 Skin rash **Criterion** 21 Hypothermia—hyperthermia **Criterion** 22 Chemicals and gasses **Criterion** 23 Convulsions **Criterion** 24 Abdominal and back pain **Criterion** 25 possible death **Criterion** 26 Reduced consciousness **Criterion** 27 Psychiatry -suicidal **Criterion** 28 Respiratory difficulty **Criterion** 29 Alcohol poisoning and overdose **Criterion** 30 Child with symptoms **Criterion** 31 Extremity pain **Criterion** 32 Traffic accident **Criterion** 33 Accident **Criterion** 34 Urinary tract **Criterion** 35 Violence **Criterion** 36 ear, nose, throat **Criterion** 37 eyes

*Translated from Danish Index for emergency care, country version 1,8. Revised July 2017.^1^

## Abbreviations

CPAP: Continuous positive airway pressure
EMS: Emergency Medical Services
ICD: Implantable Cardioverter Defibrillator
PEPR: Prehospital electronic patient record
RRV: Rapid response vehicle
GCS: Glasgow Coma Scale
